# Elevated YKL-40 expression is associated with a poor prognosis in breast cancer patients

**DOI:** 10.18632/oncotarget.14280

**Published:** 2016-12-27

**Authors:** Guoxing Wan, Longchao Xiang, Xue Sun, Xuanbin Wang, Hongliang Li, Wei Ge, Fengjun Cao

**Affiliations:** ^1^ Department of Oncology, Renmin Hospital, Hubei University of Medicine, Shiyan 442000, Hubei, China; ^2^ Laboratory of Wudang Local Chinese Medicine Research, Hubei University of Medicine, Shiyan 442000, Hubei, China; ^3^ Laboratory of Chinese Herbal Pharmacology, Oncology Center, Renmin Hospital, Hubei University of Medicine, Shiyan 442000, Hubei, China; ^4^ Department of Oncology, Renmin Hospital, Wuhan University, Wuhan 430060, Hubei, China

**Keywords:** YKL-40, breast cancer, prognosis, meta-analysis

## Abstract

Numerous studies have investigated the prognostic role of YKL-40 in breast cancer, but yielded inconsistent results. To derive a more precise evaluation, relevant publications assessing the association between YKL-40 expression and clinical outcome of breast cancer patients were electronically searched and identified. A combined analysis of included studies was performed using fixed- or random-effect model to calculate the pooled hazard ratio (HR) or odds ratio(OR) and 95% confidence interval (95%CI) for the assessment of the association. Ten eligible studies involving 1250 patients were ultimately included in the meta-analysis. Overall, the pooled analysis showed that elevated YKL-40 expression was significantly associated with a poor overall survival(OS: HR=1.48, 95%CI= 1.11-1.97) and disease-free survival(DFS: HR=1.51, 95%CI= 1.10-2.07). The subgroup analysis by detection methods revealed an unfavorable OS in breast cancer patients with elevated YKL-40 expression evaluated by IHC(HR=1.39, 95%CI=1.12-1.71) but not by ELISA/RIA. Also, the stratification analysis by ethnicity showed a significant association between increased YKL-40 expression and shorter OS of breast cancer patients in western population(HR=1.51, 95%CI=1.03-2.21) as well as Asian population (HR=1.40, 95%CI= 1.05-1.86). Similarly, the subgroup analysis by detection methods revealed a significantly inferior DFS in breast cancer patients with increased YKL-40 expression disregarding the use of IHC(HR=2.02, 95%CI=1.47-2.79) or ELISA/RIA(HR=1.06, 95%CI= 1.02 -1.10). Additionally, increased YKL-40 expression was found to significantly correlate with larger tumor size (OR=2.38, 95%CI=1.41-4.05).The present meta-analysis indicate that elevated YKL-40 expression is associated with a poor prognosis in breast cancer patients. YKL-40 may serve as a promising predictive biomarker of prognosis of breast cancer.

## INTRODUCTION

Breast cancer remains the most frequently diagnosed cancer among women worldwide and accounts globally for the leading cause of cancer death in females, taking up 29% of all new cancer cases and 14% of all cancer deaths [1, 2]. Although the clinical outcome presenting a 5-year survival rate of 95% has been gradually improved with advances in treatments over decades, the disease continues to be devastating for its suffers particularly at risk, forwarding the paramount necessity and importance to discriminate prognosis and optimize treatment strategy [3]. Presently, the prognostication and treatment for breast cancer relied primarily on several traditionally confirmed prognostic factors including tumor stage (TNM), tumor histological grade and lymph node status, as well as estrogen receptor (ER), progesterone receptor (PR), and human epidermal growth factor (EGF) receptor 2 (HER2) [4]. However, the discriminant value of most potential prognostic predictors remains partially insufficient to the optimal therapeutic course for different individuals, which leaves much to be desired in identifying more reliable and clinically applicable biomarkers for prognosis.

YKL-40 also known as human cartilage glycoprotein-39 or chitinase-3-like-1, is a phylogenetically conserved heparin- and chitin-binding glycoprotein without chitinase activity [5]. YKL-40 belongs to a group of mammalian proteins with an amino acid sequence similar to the 18-glycosyl hydrolase group of bacterial chitinases [6, 7], and is found to be expressed and secreted by chondrocytes, synoviocytes, hepatic stellate cells, vascular smooth muscle cells, neutrophils, while it commonly overexpressed in several types of cancer including breast, colon, kidney, lung, ovarian, prostate, uterine, osteosarcoma, glioblastoma and germ cell tumors [8, 9]. Biologically, the exact function of YKL-40 remains largely unknown, while it is currently evidenced that YKL-40 may have been implicated in proliferation of chondrocytes and fibroblasts, differentiation of macrophage, migration and reorganization of vascular endothelial cells as well as inflammation and remodeling of extracellular matrix [10]. Reportedly, in addition to the roles mentioned above, inhibition of YKL-40 was found to be able to attenuate the tube formation of microvascular endothelial cells in vitro and suppresse tumor growth, angiogenesis, and progression of brain tumors [11]. Such biophysiological activities were also observed in several other malignancies [12], suggesting its angiogenic properties in cancer development. Clinically, YKL-40 is found to be expressed in serum and tumor tissue in patients. Recently, aberrantly elevated expression of YKL-40 has been observed in a number of human malignancies, such as breast, ovarian, prostate, colon, CNS, bone, and skin cancers in several independent studies [13]. Of note, it is reported that serum levels of YKL-40 in patients with glioma are related with tumor grade and burden [14], and high YKL-40 expression is associated with poor survival of patients with lung cancer, glioblastoma, colorectal cancer, hepatocellular carcinoma, gastric cancer [15–18], supporting the notion that elevated YKL-40 expression may serve as a useful potential biomarker of prognosis for cancer patients.

Numerous studies have evaluated the association between YKL-40 expression and survival of patients with breast cancer, however, the results were still inconsistent. Some studies demonstrated that high YKL-40 expression was associated with poor prognosis in breast cancer patients [19, 20], but others failed to confirm such association [3, 21], which may be due to several reasons. For example, the detection methods for YKL-40 expression varied among studies, or the smaller sample size of an individual study was underpowered to reflect the exact role, or the ethnicity variation potentially affected the results. Accordingly, we conducted a meta-analysis with all available published studies to determine the prognostic role of YKL-40 expression in breast cancer.

## RESULTS

### Search results and study characteristics

**Figure 1:**
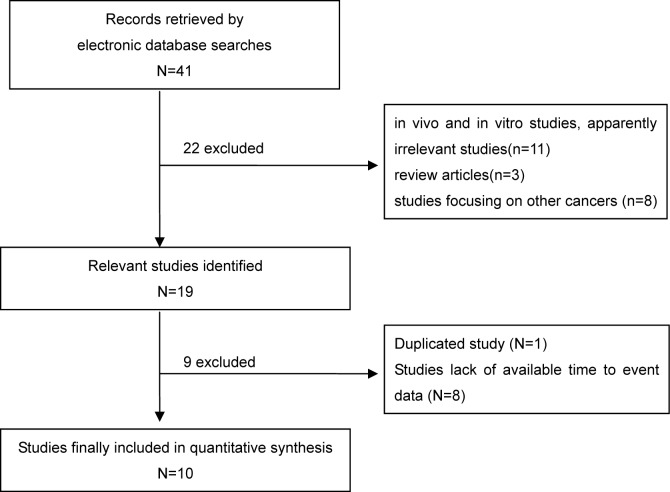
Flow chart of study selection for the pooled analysis

A total of forty-one articles were initially retrieved according to the established search strategy, while ten studies involving 1250 patients were ultimately included in the meta-analysis [3, 19–27], with individual samples ranging from 30 to 399 (Table 1). The flow chart of study selection was detailed in Figure 1. Of which, nine studies were reported in English and one reported in Chinese. Moreover, there were seven studies conducted in western populations and three studies conducted in Asian populations. The level of YKL-40 expression was determined by immunohistochemistry (IHC) in six studies, while by radio immunity assay (RIA) in two studies and by enzyme-linked immuno sorbent assay(ELISA) in two studies. The evaluation of YKL-40 expression was defined according to the percent and/or intensity of YKL-40 staining in tumor cells by IHC as well as a determinate cut-off value of serum YKL-40 level by RIA or ELISA. Of the ten included articles, adjusted multivariate analyses for the association between YKL-40 expression and the survival outcome of breast cancer patients were conducted in six publications, while unadjusted univariate analyses for this association were performed in four publications. Eventually, eight individual studies reporting the relevant HRs with 95%CIs on OS and six individual studies concerning the association regarding DFS were enrolled. The main characteristics of the studies enrolled in the meta-analysis are summarized in Table 1. According to the Newcastle–Ottawa Scale, the scores of quality assessment for all included studies ranged from six to eight triangles, suggesting a good quality (Supplementary Table S1).

**Table 1:** Main characteristics of the included studies in the meta-analysis

First author, year	Country	Ethnicity	Study design	Detection method	Cut-off	Stage	Sample size	Chemotherapy scheme	Adjusting variables	HR(95%CI)	HR(95%CI)
										DFS	OS
Shao,2011[3]	USA	Western	retrospective	IHC	percent and intensity	I-III	79	NR	NA	-	1.60(0.85-3.01)
Jensen,2003[19]	Denmark	Western	prospective	RIA	High(>168ug/liter)	IV	30	Anthracycline-based	HER2 and ER status	1.96(1.20-3.20)	2.57(1.60-4.10)
Julia,2003[22]	Denmark	Western	prospective	ELISA	High(>10ug/l)	I-III	271	CMF	lymph node status, ER status,age,tumor size, and menopausal status.	1.73(1.03-2.91)	1.77(1.03-3.06)
Wang,2012[23]	China	Asian	prospective	IHC	percent and intensity	I-III	120	NR	tumor size,stage,Pathological classification,Lymph node metastasis	-	1.60(0.40-6.34)
Kim,2007[20]	USA	Western	prospective	IHC	percent	I-IV	109	NR	NA	1.06(1.01-1.10)	-
Johansen,1995[24]	USA	Western	retrospective	RIA	High(>207ug/l)	IV	60	CMF	Serum,BGP,ASAT,LDH,AP,Hb	2.99(1.42-6.29)	-
Yamac,2008[25]	Turkey	Western	prospective	ELISA	High(>149.5ug/l)	I-III	45	CAF	lymph node status, ER and PR status,tumor size and tumor grade.	-	1.004(1.00-1.07)
Roslind,2008[21]	Denmark	Western	prospective	IHC	intensity	I-III	399	CMF or CEF	treatment, menopausal status,tumor size, nodal status,histological type and grade,steroid hormone receptor status, HER2 status and TOP2A status	1.22(0.87–1.72)	1.31(0.91–1.87)
Kang,2014[26]	Korea	Asian	retrospective	IHC	intensity	I-III	77	NR	NA	1.72(0.46-6.45)	2.19(0.50-9.66)
Zhai,2012[27]	China	Asian	prospective	IHC	percent and intensity	I-III	60	NR	NA	-	1.361(1.01-1.83)

IHC, immunohistochemistry; RIA, Radio Immunity Assay; ELISA, Enzyme-Linked Immuno Sorbent Assay; NR, not reported; NA, not available; DFS, disease-free survival; OS, overall survival

### Meta-analysis

**Figure 2:**
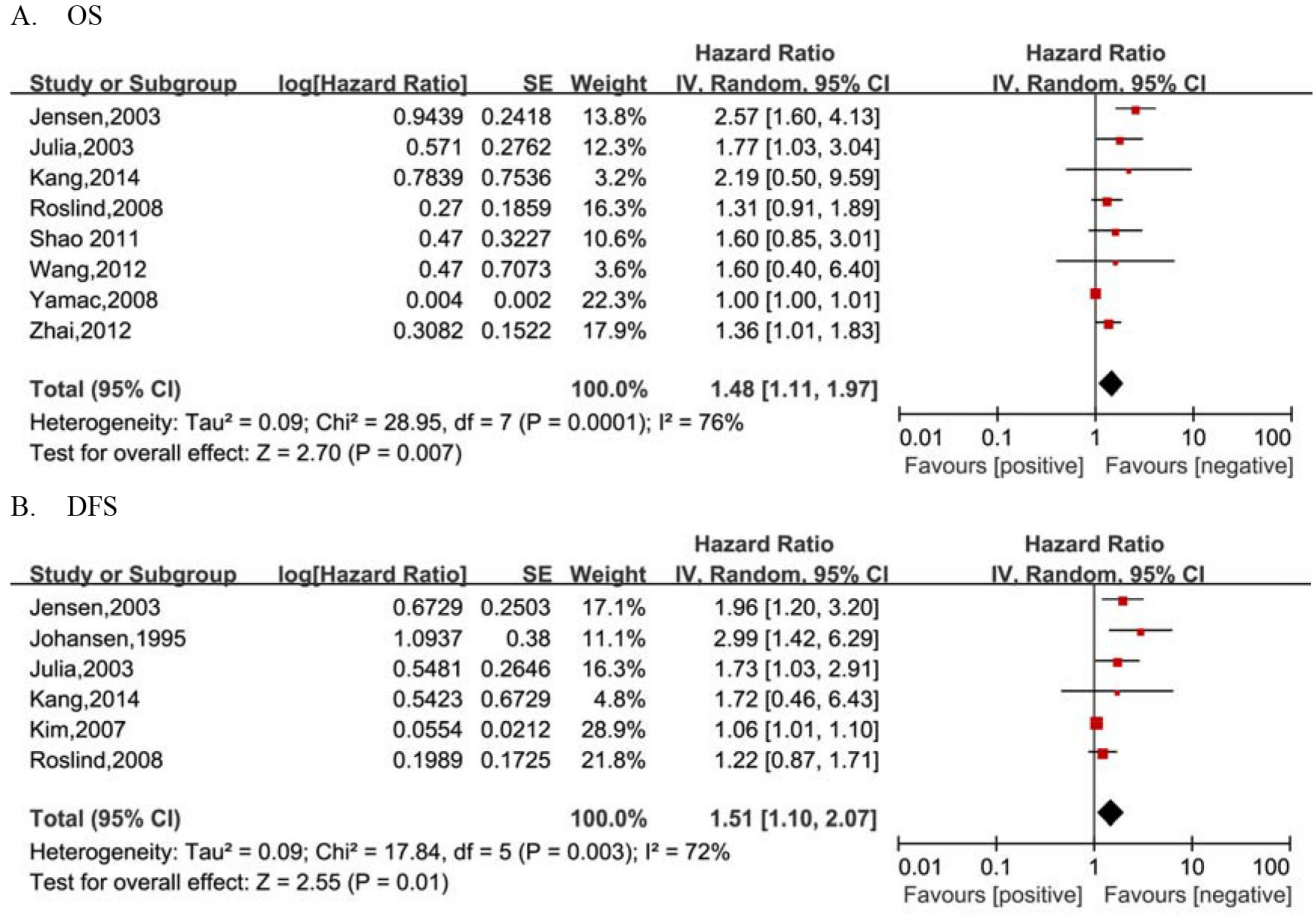
Forest plots of studies evaluating pooled hazard ratio (HR) and 95% confidence interval 95% CI) of YKL-40 for breast cancer survival **A.** Overall survival(OS). **B.** Diseaase- free survival(DFS).

Overall, as noted in Figure 2 and Table 2, the pooled analyses suggested that elevated YKL-40 expression was significantly associated with a poor prognosis regarding OS(HR=1.48, 95%CI= 1.11-1.97) and DFS(HR=1.51, 95%CI=1.10-2.07) in patients with breast cancer in the random -effect model, despite the presence of heterogeneity among the studies (OS: *P*_h_=0.0001, I^2^=76%; DFS: *P_h_*=0.003, I^2^=72%).

**Table 2:** Meta-analysis results of the association between YKL-40 expression and survival of breast cancer patients

Survival	Subgroup		Number of Pooled analyses		*P* value	Heterogeneity		Effect model
			studies	HR(95%CI)		P_h_	I^2^	
DFS	Overall		6	1.51 [1.10, 2.07]	**0.01**	0.003	72%	R
	Detection methods	IHC	3	2.02 [1.47, 2.79]	**<0.0001**	0.49	0%	F
		ELISA/RIA	3	1.06 [1.02, 1.10]	**0.006**	0.55	0%	F
OS	Overall		8	1.48 [1.11, 1.97]	**0.007**	0.0001	76%	R
	Detection methods	IHC	5	1.39 [1.12, 1.71]	**0.002**	0.95	0%	F
		ELISA/RIA	3	1.60 [0.84, 3.07]	0.15	<0.0001	95%	R
	Ethnicity	Asian	3	1.40 [1.05, 1.86]	**0.02**	0.81	0%	F
		Western	5	1.51 [1.03, 2.21]	**0.04**	0.0001	83%	R

IHC, Immunological Histological Chemistry; RIA, Radio Immunity Assay; ELISA, Enzyme-Linked Immuno Sorbent Assay; DFS, disease-free survival; OS, overall survival; HR, hazard ratio; 95%CI, 95% confidence interval; P_h_, p value of the Q test for heterogeneity. R, random-effect model; F, fixed-effect model.

Considering the obvious heterogeneity across studies, the subgroup analysis regarding OS based on detection methods was performed to explore the source of heterogeneity and the results revealed that elevated YKL-40 expression had a significantly poor OS effect on breast cancer patients both by IHC(HR=1.39, 95%CI=1.12-1.71) but not by ELISA/RIA(HR=1.60, 95%CI= 0.84-3.07), with a reduced heterogeneity. Moreover, the stratification analysis by ethnicity showed a significant association between increased YKL-40 expression and shorter OS of breast cancer patients in western population(HR=1.51, 95%CI=1.03-2.21) as well as Asian population (HR=1.40, 95%CI=1.05-1.86). Likewise, the subgroup analyses by detection methods revealed a significantly inferior DFS in breast cancer patients with increased YKL-40 expression disregarding the use of IHC(HR=2.02, 95%CI=1.47-2.79) or ELISA/RIA(HR=1.06, 95%CI= 1.02 -1.10). Meanwhile, the heterogeneity across studies was significantly reduced, a fixed-effect model were therefore applied in the subgroup analyses (Table 2).

**Figure 3:**
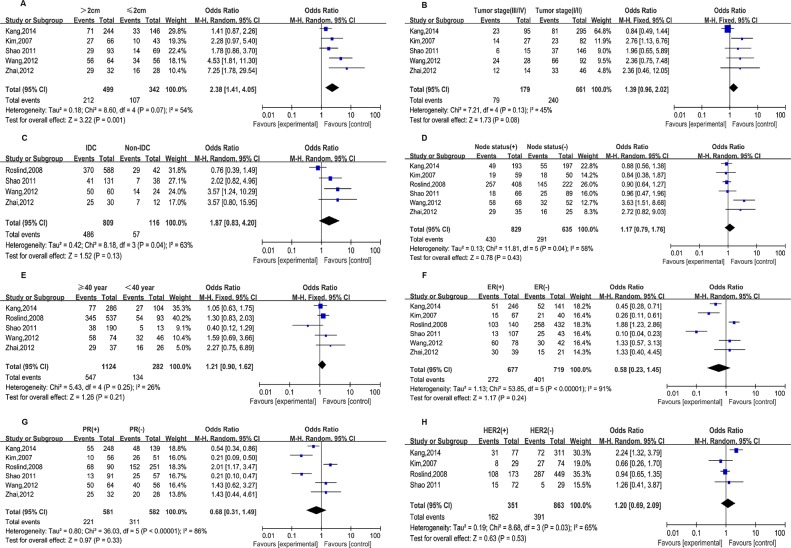
Forest plots of studies evaluating the association between YKL-40 expression and clinicopathological parameters in breast cancer **A.** tumor size. **B.** tumor stage. **C.** tumor histology. **D.** node status. **E.** age. **F.** ER status. **G.** PR status. **H.** HER2 status. IDC, invasive ductal carcinoma.

Moreover, the associations between YKL-40 expression and clinicopathological parameters of breast cancer were also investigated, however, only two included studies (Julia,et al, [22] and Yamac,et al, [25]) which detected the YKL-40 expression by ELISA/RIA reported the concerned association. Therefore, six studies detecting the YKL-40 expression by IHC were enrolled in the pooled analyses. As illustrated in Figure 3, increased YKL-40 expression was found to significantly correlate with larger tumor size (OR=2.38, 95%CI=1.41-4.05, *P*=0.001). Furthermore, a boardline association was shown between elevated YKL-40 expression and advanced tumor stage (OR=1.39, 95%CI=0.96-2.02, *P*=0.08). Regretfully, similar association was not observed regarding tumor histology, node status, age, ER, PR and HER2 status.

### Publication bias and sensitivity analysis

**Figure 4:**
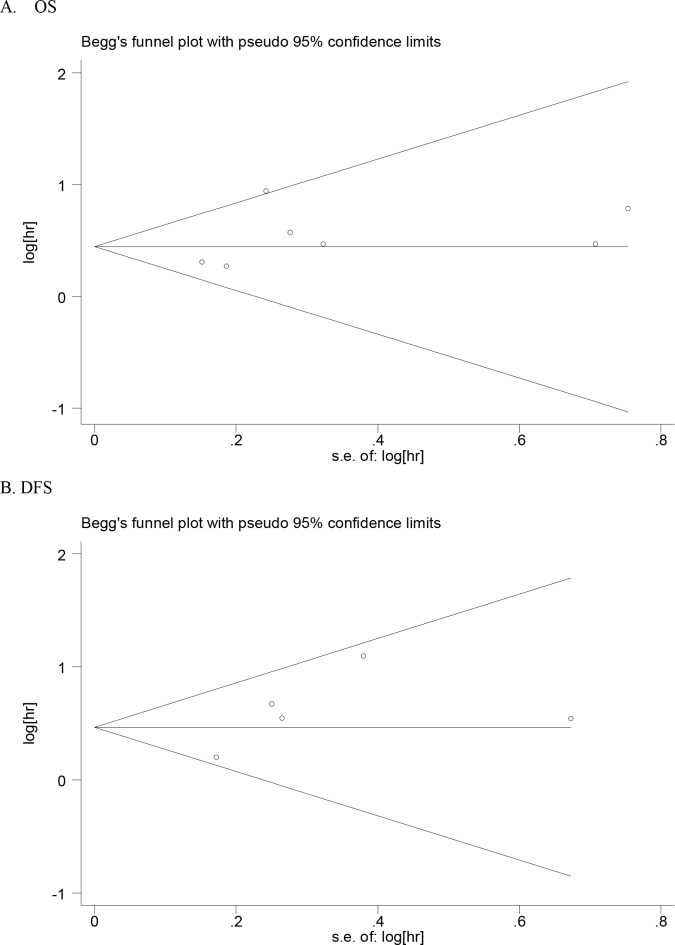
Funnel plots for the assessment of publication bias in this study **A.** Overall survival(OS). **B.** Diseaase- free survival(DFS).

Begg’s funnel plot and Egger’s test were adopted to assess potential publication bias in the current meta-analysis. As a result, a visual asymmetry was observed in the funnel plots concerning the effect of YKL-40 expression on OS and DFS of breast cancer patients, which was also presented by Egger’s test(OS: p=0.004; DFS: p=0.015), suggesting the presence of potential publication bias in the meta-analysis. However, as shown in Table 3 and Figure 4, the observed publication bias disappeared with a significant reduction of heterogeneity in the analysis regarding OS by omitting the study by Yamac et al. [25] and regarding DFS by the omission of the study by Kim et al. [20], respectively, suggesting that the omitted studies may contribute significantly to the evident heterogeneity. To examine the stability of the overall results, the sensitivity analysis by sequential omission of individual studies was performed and the significance of pooled results for OS and DFS was not substantially altered, indicating the robustness of the results (Table 3).

**Table 3:** Sensitive analyses results of the associations of YKL-40 expression with DFS and OS in breast cancer patients

Survival	Exclusion of studies	Pooled analyses	*P* value	Heterogeneity		Effect model	Egger’s test
		HR(95%CI)		P_h_	I^2^		*p*
DFS	Kim,2007 [20]	1.56 [1.30, 1.87]	**<0.0001**	0.38	6%	F	0.354
OS	Yamac,2008 [25]	1.59 [1.27, 2.00]	**<0.0001**	0.21	32%	F	0.228

DFS, disease-free survival; OS, overall survival; HR, hazard ratio; 95%CI, 95% confidence interval; *P_h_*, p value of the Q test for heterogeneity. R, random effect model; F, fixed effect model.

## DISCUSSION

Previous studies investigating the influence of YKL-40 expression on clinical outcome of patients with breast cancer have reported varying even conflict results, and there is still lack of high-quality evidence for YKL- 40 as a molecular biomarker in breast cancer patients. Currently, we focused on the prognostic utility of YKL-40 in breast cancer with a comparatively large sample size using a powerful approach, providing more reliable evidence. To our knowledge, the present study is the first to reevaluate the association between YKL-40 expression and the clinical outcome of breast cancer patients by meta-analysis, demonstrating a inferior impact of elevated YKL-40 expression on the OS as well as DFS, which suggests its prognostic significance as a potentially serviceable biomarker.

It is reported that YKL-40 is overexpressed in the adenocarcinomas of breast, colon, ovarian, uterine, prostate, kidney, and lung by the search of YKL-40 protein sequence against the dbest database at the National Center for Biotechnology Information using the BLAST program [19]. Accumulative evidence have also demonstrated an association of elevated YKL-40 expression with poor prognosis in patients with breast, colorectal cancer, lung and other types of cancers [28]. However, the underlying mechanism remains to be clearly elucidated. Progressively, YKL-40 has recently been shown to exhibit effective growth factor activity involved in tumor development and inflammation processes [9]. Several experimental models supported the notion that YKL-40 might play a stimulative role in tumor initiation through binding to RAGE [29], and might be able to induce the proliferation of cancer cell via ERK1/2 [30]. Moreover, YKL-40 was likely to promote tumor angiogenesis by interacting with syndecan-1 on endothelial cells as well as metastasis by stimulating production of pro-inflammatory and pro-invasive factors such as MMP-9, CCL2 and CXCL2 [31, 32]. Furthermore, YKL-40 was considered a potential modulator of inflammatory tumor microenvironment by inducing production of pro- and anti-inflammatory cytokines and chemokines [33]. Correspondingly, targeting YKL-40 by neutralizing antibodies exerted anti- cancer effect in preclinical animal model [34]. Along with these findings, YKL-40 was suggested to be used as a promising predictive biomarker of cancer outcome in combination with other circulating factors and might serve as an attractive candidate for tumor therapy and immunomodulation [35]. Nevertheless, the prognostic significance of YKL-40 in breast cancer patients remains undetermined due to the discrepant reports by studies focusing on the association between YKL-40 expression and survival of patients with breast cancer, which may be partially attributed to varying sample sizes, ethnicities and evaluation methods of YKL-40 expression in individual studies.

In the present study, ten publications involving 1250 patients with breast cancer were combined to yield statistics with more power, indicating a significant association of YKL-40 overexpression with poor OS and DFS in breast cancer patients, which was in keeping with the findings regarding glioblastoma by a similar approach [36]. In light of the potential impact of variations in ethnicities and evaluation methods of YKL-40 expression on the combined results, the subgroup analyses focusing on OS were performed based on ethnicity and detection methods as well. Consequently, elevated YKL-40 expression was found to be associated with a poor OS in Asian as well as western populations, indicating little influence of ethnicity variation on the concerned association. However, such association was only well-confirmed in studies using IHC to evaluate YKL-40 expression but not in studies with ELISA/RIA methods with evident heterogeneity across individual studies. Considering the inconsistent trend of results and significant heterogeneity in the subgroups, a lack of uniform detect methods and evaluation criteria may be a restriction for a pooled analysis to confidently illustrate the prognostic significance of YKL-40 for OS in breast cancer. Additionally, the analyses regarding DFS stratified by detection methods revealed was also conducted and revealed an unfavorable DFS in breast cancer patients with YKL-40 overexpression evaluated both by IHC and by ELISA/RIA without observed heterogeneity. These findings suggested that YKL-40 might be more appropriate to serve as a biomarker of DFS. Of note, we did not perform subgroup analysis by ethnicity since all the studies were conducted in western populations except for one study conducted in Asian population. Besides, increased YKL-40 expression was suggested to potentially accelerate the tumor size and advanced stage, which also supported the unfavorable prognostic role of YKL-40 overexpression in breast cancer. However, the present study failed to confirm similar association regarding tumor histology, node status, age, ER, PR and HER2 status, possibly due to the limited studies included. Nevertheless, the considerable heterogeneity across studies when examining the correlation between YKL-40 expression and prognosis and clinicopathological parameters should be noticed as well, which implied that caution should be held in the appropriate interpretation of our findings.

For every plus, there is a minus. As a meta-analysis, the present study allows us to get a better understanding on the prognostic role of YKL-40 expression in breast cancer patients by increasing the statistical power through combining data from numerous studies, however, several limitations in the meta-analysis should not be neglected as well. Firstly, potential publication bias is a major concern in the meta-analysis although it disappears by the omission of a study and the heterogeneity across studies was significantly reduced, the results were supposed to be interpretated with caution. Secondly, the estimated HRs with corresponding 95%CIs for survival data were obtained from univariate analysis or multivariate analysis with different adjustment variables, or calculated by Kaplan–Meier curves, which may limited the reliability of results. Thirdly, hormone status is a relatively important factor relevant to the treatment and prognosis of breast cancer [19], however, few studies investigating the association were limited to a specific subtype of breast cancer. Fourthly, in the assessment of biomarkers, the use of a standard threshold is of great importance. The differences in cut-off values for elevated YKL-40 expression may contribute to the observed heterogeneity. Lastly, combining data from different heterogeneous studies through meta-analysis may limit the generality of the pooled findings since patients in individual studies received different types of treatments. Given the above limitations, caution should thereby be utilized when interpreting these results in the meta-analysis.

In conclusion, the present meta-analysis suggests that elevated YKL-40 expression is markedly associated with worse survival outcome in breast cancer patients, implying that YKL-40 may be a promising predictive biomarker of prognosis in breast cancer patients. Considering the limitations, well-designed and multicenter randomized controlled trials with uniform evaluation standard of the biomarker and study populations with similar clinical characteristics and managements are warranted to further confirm the results.

## MATERIALS AND METHODS

### Search strategy and study selection

A systematic literature search was performed electronically to identify relevant studies regarding the association of YKL-40 expression with survival in breast cancer patients in PubMed, Embase, Google Scholar, Cochrane Library database, Web of Science, Wanfang, China National Knowledge Infrastructure and China Biomedical Literature Database up to June 2016, which was restricted to human studies published in either English or Chinese. We developed a search strategy using the following search terms: “breast cancer or breast carcinoma” and “YKL-40 or CHI3L1 or chitinase 3-like-1” in all possible combinations. Concurrently, the reference lists of retrieved publications were perused manually to check for additional eligible studies.

### Selection criteria

Studies were consider eligible if they satisfied the following inclusion criteria: (1) studies assessing the association between YKL-40 expression and clinicopathological parameters, overall survival(OS) or disease-free survival(DFS, including progression-free survival or recurrence-free survival) in breast cancer patients; (2) studies directly or indirectly providing available information on survival data by the Kaplan-Meier curve or available reports of clinical outcome to estimate the hazard ratio (HR) with the corresponding 95% confidence interval (95% CI); (3) all cancer patients pathologically confirmed. Studies were excluded if they met the following criteria: (1) overlapped studies; (2) insufficient data on outcome or the HR/OR and the corresponding 95%CI unable to be obtained directly or indirectly; (3) review articles, meta-analysis, case reports, or letters. In case several publications reported the concerned association in a same study population, only the most informative study was adopted.

### Data extraction and quality assessment

The following information was evaluated and extracted independently by two investigators according to a unified standard aforehand proposed: first author, publication year, country, ethnicity, study design, detection methods of YKL-40 expression, cut-off value of YKL-40 expression evaluation, tumor stage, tumor size, node status, tumor histology, age, ER, PR, HER2 status, sample size, chemotherapy scheme, adjusting variables, the HRs and the corresponding 95%CIs for OS or DFS. For the observational studies, the estimates of HRs and the corresponding 95%CIs from both unadjusted and adjusted models were likely used. However, if the estimates by univariate analysis and multivariate analysis were both reported in the same article, the latter was preferably selected. The quality assessment of included studies was performed according to the Newcastle Ottawa Scale [37], concerning several of important factors as previously described [36]. All the analyses were based on previously published studies, thus no ethical approval or patient consent was required. All discrepancies were discussed and resolved until a consensus was reached.

### Statistical analysis

Individual HRs or ORs and associated 95%CIs were pooled to evaluate the impact of YKL-40 expression on the survival or clinicopathological parameters of breast cancer patients, which was performed under a fixed- or random-effect model according to the heterogeneity across the studies with Review Manager version 5.2 software (The Cochrane Collaboration, Oxford, UK) and Stata SE12.0 (Stata Corporation, TX, USA). Some HRs and their 95% CIs which were not directly reported by the original studies were estimated by available data or Kaplan–Meier curves using the methods reported by Tierney et al [38]. and Parmar et al. [39]. The significance of the pooled HR or OR was determined by Z test and p<0.05 was considered significant. Between-study heterogeneity was evaluated by the Cochran’s χ2-based Q test and the I-squared test, and p>0.10 or I^2^ < 50% indicated no obvious heterogeneity [40], then the fixed- effect model was applied to calculate the pooled HR or OR [41], otherwise, the random-effect model was utilized [42]. Also, we performed subgroup analyses by some potentially important confounding variables such as ethnicities of study population and detection methods of YKL-40 expression. The Begg’s funnel plot was used to visually assess the potential publication bias [43], which was further examined by Egger’s linear regression test (p<0.05 was considered statistically significant) [44]. Moreover, the sensitivity analysis by sequential omission of individual studies was also conducted to assess the stability of results.

## SUPPLEMENTARY TABLE


