# Supplementary material for: Elevated YKL-40 expression is associated with a poor prognosis in breast cancer patients
Source: Oncotarget. 2016 Dec 27;8(3):5382–91. doi: 10.18632/oncotarget.14280 (PMC5354916; doi:10.18632/oncotarget.14280)
Supplement: Supplementary file 1 [file oncotarget-08-5382-s001.pdf]

Elevated YKL-40 expression is associated with a poor prognosis in breast cancer patients

SUPPLEMENTARY TABLE

Supplementary Table S1: The quality assessment of the included studies appraised in reference to the Newcastle-Ottawa statement

First author, year	Country	Selection (4)				Comparability (2)		Outcome (3)			Total
Shao,2011 [3]	USA	▲	▲	▲		▲		▲	▲		6
Jensen,2003 [19]	Denmark	▲	▲			▲	▲	▲	▲	▲	7
Julia,2003 [22]	Denmark	▲	▲	▲		▲	▲	▲	▲	▲	8
Wang,2012 [23]	China	▲	▲	▲		▲	▲	▲	▲		7
Kim,2007 [20]	USA	▲	▲			▲	▲	▲	▲		6
Johansen,1995 [24]	USA	▲	▲			▲	▲	▲	▲		6
Yamac,2008 [25]	Turkey	▲	▲	▲		▲	▲	▲	▲		7
Roslind,2008 [21]	Denmark	▲	▲	▲	▲	▲	▲	▲	▲	▲	9
Kang,2014 [26]	Korea	▲	▲			▲	▲	▲	▲		6
Zhai,2012 [27]	China	▲	▲			▲	▲	▲	▲		6
